# Serine to proline mutation at position 341 of MYOC impairs trabecular meshwork function by causing autophagy deregulation

**DOI:** 10.1038/s41420-024-01801-1

**Published:** 2024-01-11

**Authors:** Xuejing Yan, Shen Wu, Qian Liu, Ying Cheng, Yufei Teng, Tianmin Ren, Jingxue Zhang, Ningli Wang

**Affiliations:** 1 Beijing Institute of Ophthalmology, Beijing Tongren Eye Center, Beijing Tongren Hospital, Capital Medical University; Beijing Ophthalmology & Visual Sciences Key Laboratory, Beijing, 100730 China; 2 Beijing Institute of Brain Disorders, Collaborative Innovation Center for Brain Disorders, Capital Medical University, Beijing, 100069 China

**Keywords:** Cell biology, Autophagy

## Abstract

Glaucoma is a highly heritable disease, and myocilin was the first identified causal and most common pathogenic gene in glaucoma. Serine-to-proline mutation at position 341 of myocilin (MYOC^S341P^) is associated with severe glaucoma phenotypes in a five-generation primary open-angle glaucoma family. However, the underlying mechanisms are underexplored. Herein, we established the MYOC^S341P^ transgenic mouse model and characterized the glaucoma phenotypes. Further, we systematically explored the functional differences between wild-type and MYOC^S341P^ through immunoprecipitation, mass spectrometry, and RNA-seq analyses. We found that MYOC^S341P^ transgenic mice exhibit glaucoma phenotypes, characterized by reduced aqueous humor outflow, elevated intraocular pressure, decreased trabecular meshwork (TM) cell number, narrowed Schlemm’s canal, retinal ganglion cell loss, and visual impairment. Mechanistically, the secretion of dysfunctional MYOC^S341P^ accumulated in the endoplasmic reticulum (ER), inducing ER stress and dysregulation of autophagy, thereby promoting TM cell death. We describe an effective transgenic model for mechanistic studies and the screening of therapeutic targets. Our data generated from high-throughput analyses help elucidate the mechanism underlying mutant MYOC-related glaucoma.

## Introduction

Glaucoma, a leading cause of irreversible vision loss, is predicted to affect more than 112 million people by 2040 [1]. Primary open-angle glaucoma (POAG) is the most common type of glaucoma, characterized by retinal ganglion cell (RGC) loss and visual impairment [2]. Elevated intraocular pressure (IOP) is the leading risk factor for, and the only modifiable feature of, POAG [3].

The maintenance of normal IOP is dependent on the balance between aqueous humor inflow and outflow [4]. The trabecular meshwork (TM) is the main structure responsible for the regulation of aqueous humor circulation, and its dysfunction induces aqueous humor outflow resistance. Hence, TM dysfunction is linked to elevated IOP [5]. Many factors can cause TM damage, including fibrosis [6], hypophagocytosis [7], extracellular matrix deposition [8], and decreased TM cell number [9]. However, the molecular mechanisms underlying TM damage are underexplored.

Hundreds of genomic loci have been associated with POAG by genome-wide association studies [10, 11], but only a few genes are validated to be correlated with TM dysfunction, including *LOXL4*, *CYP1B1*, *GLIS1*, and *MYOC* [12–16]. *MYOC* was the first gene identified as causally linked to POAG, and some mutations of *MYOC* cause TM damage [17, 18].

It has been reported that the severity of glaucoma in patients carrying different MYOC mutations varies. Clinically, Q368X represents less severe and late-onset POAG [19], whereas POAG patients carrying a G367R [20–22] or P370L [23, 24] mutation are more severe and early-onset disease. Mechanically, P370L impairs TM function mainly by perturbing ER and mitochondrial function [25–27]. Nevertheless, studies showed that some MYOC mutations lead to glaucoma phenotypes [28–30], while certain transgenic mice expressing mutated *MYOC* genes do not develop glaucoma [31]. So, the different mutations in MYOC may play diverse roles in the pathogenesis of glaucoma and potential clinical therapy. Our previous studies showed that N450Y or P370L mutation in MYOC leads to glaucoma phenotypes in transgenic mice models [15, 27]. Moreover, S341P mutation of the MYOC gene (MYOC^S341P^) is associated with a severe pathological glaucoma phenotype in five generations of Chinese families [32]. Studies on the pathogenesis of mutant MYOC have mainly focused on cellular aggregation-induced ER stress [17, 18], and ER stress-mediated autophagy activation is believed to participate in TM injury [33]. Hitherto, it remains unclear whether MYOC^S341P^ mutation has pathogenic effects on TM or if autophagy is involved in TM injury caused by MYOC^S341P^.

Autophagy is a process in which eukaryotic cells use lysosomes to degrade cytoplasmic proteins and damaged organelles under the regulation of autophagy-related genes [33]. It is essential to maintain cellular homeostasis and counter different cellular stresses. Wild-type MYOC (MYOC^WT^) proteins are degraded by ubiquitin-proteasome and lysosomal pathways, but in the case of certain mutant MYOC proteins, autophagy is induced to deal with cellular aggregation [15, 16, 34]. Different mutant MYOC proteins vary in their susceptibility to intracellular degradation [34], indicating that they are blocked at different stages of the secretory pathway. Hence, it is reasonable to infer that various mutated MYOC proteins differ in their effects on TM pathogenesis.

In this study, we established the MYOC^S341P^ transgenic mouse model and found that MYOC^S341P^ causes TM dysfunction and leads to glaucoma phenotypes in transgenic mice with similar characteristics to glaucoma patients. Additionally, we investigated the function of MYOC^S341P^ in two human primary TM cell lines of different origin and found that MYOC^S341P^ leads to TM cell injury. Furthermore, we showed that the biological functions of proteins that interact with MYOC^S341P^ are mainly enriched for localization to and targeting of the ER, and that MYOC^S341P^ overexpression induces ER stress. To better understand the potential underlying mechanism, we examined the changes in the expression of downstream genes using RNA-seq analysis, which demonstrated that autophagy plays a vital role. MYOC^S341P^ activates autophagy, but the process is dysregulated. Hence, our findings demonstrate that MYOC^S341P^-induced autophagy dysfunction may contribute to TM impairment and glaucoma.

## Results

### Generation of a MYOC^S341P^ mouse model

**Fig. 1:**
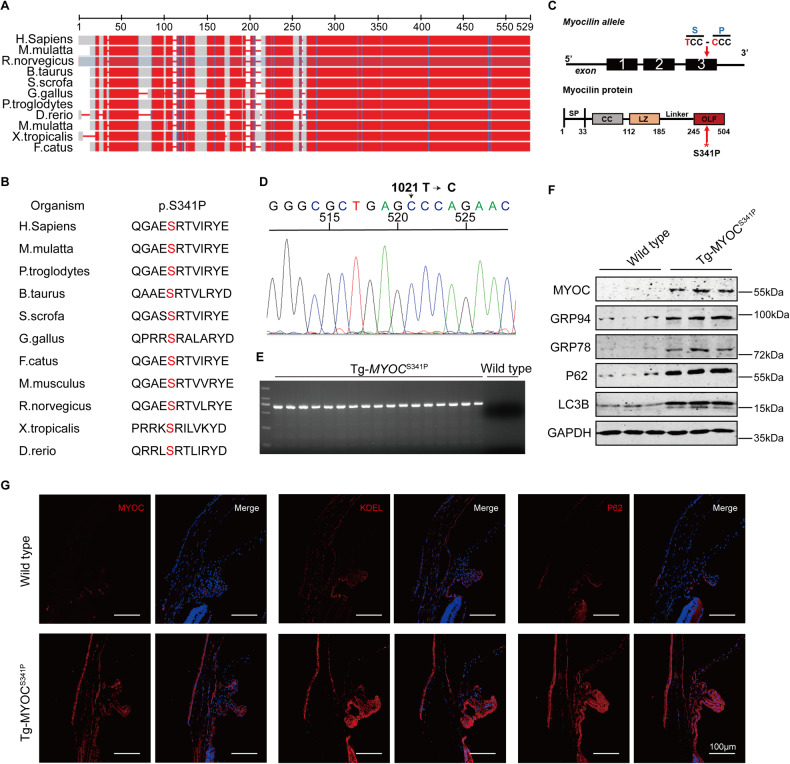
Successful construction of MYOC^S341P^ transgenic mice. **A** Alignment of MYOC protein sequences in different species. **B** Alignment of MYOC mutation site in different species. **C** MYOC gene and protein structures. The schematic diagram shows the location of the missense mutation in MYOC. **D** Sanger sequencing of genomic DNA from MYOC^S341P^ transgenic mice (*n* = 3). **E** Agarose gel electrophoresis verification of transgene integration in MYOC^S341P^ transgenic mice (*n* = 17). **F** Western blot analysis of MYOC, GRP78, GRP94, P62, and LC3B expression in anterior chamber angle tissue from MYOC^S341P^ transgenic mice (*n* = 3) and wild-type (WT) littermates (*n* = 3). **G** Immunostaining of MYOC, KDEL, and P62 in anterior chamber angle tissue in MYOC^S341P^ transgenic mice (*n* = 3) and WT littermates (*n* = 3).

The mutant S341P amino acid residue is located in the OLF domain of MYOC. It is highly conserved across species, from nematodes to higher animals (Fig. 1A–C), suggesting an important role for this locus. To verify the causative nature of MYOC^S341P^ in vivo, a MYOC^S341P^ (c.T1021C) transgenic mouse model was developed using the CRISPR/Cas9 system. Sanger sequencing and agarose gel electrophoresis were used to determine successful model construction (Fig. 1D, E). Transgene protein was overexpressed in the iridocorneal angle (Fig. 1F, G). Furthermore, ER stress and autophagy were induced in the iridocorneal angle, as indicated by western blot (Fig. 1F) and immunofluorescent staining (Fig. 1G), similar to our previous report on N450Y mutant MYOC transgenic mouse [15]. These results show that MYOC^S341P^ transgenic mice were successfully constructed.

### MYOC^S341P^ transgenic mice exhibit TM dysfunction and elevated IOP

**Fig. 2:**
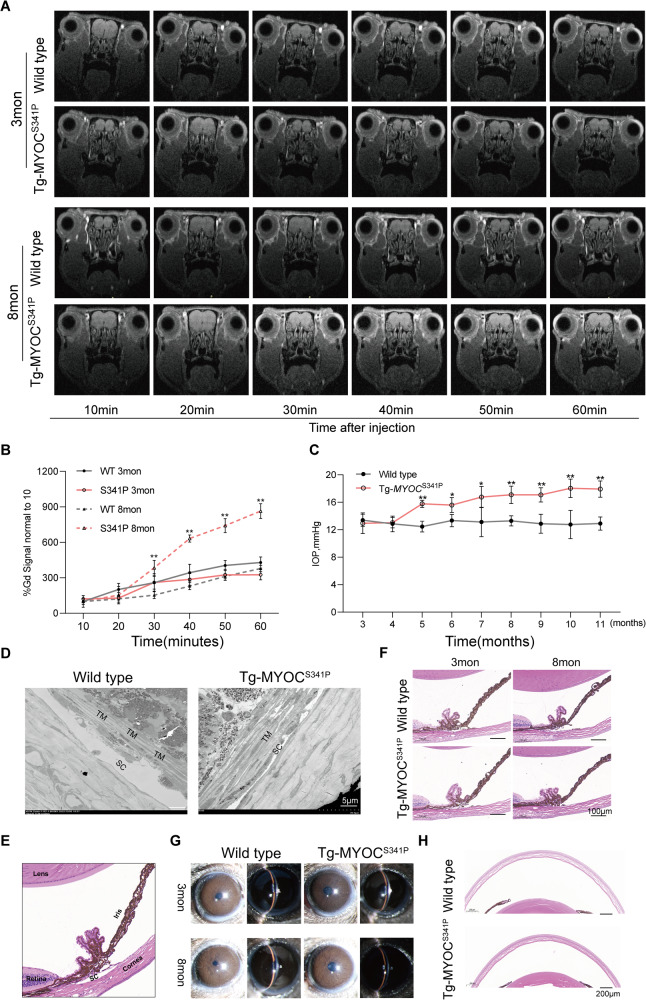
Altered aqueous humor dynamics and elevated intraocular pressure (IOP) in MYOC^S341P^ transgenic mice. **A** Gd-enhanced magnetic resonance images from MYOC^S341P^ transgenic mice and wild-type (WT) littermates at 3 and 8 months of age. 3 months: Wild-type: *n* = 6, MYOC^S341P^ transgenic mice: *n* = 6; 8 months: Wild-type: n = 6, MYOC^S341P^ transgenic mice: *n* = 6. **B** Pixel intensity was evaluated in the anterior chamber angle after Gd-DTPA injection using ImageJ software. **p < 0.01. **C** IOP was measured in MYOC^S341P^ transgenic mice (*n* = 95) and WT littermates (*n* = 66) at various ages. *p < 0.05; **p < 0.01. **D** Transmission electron microscopy analysis of trabecular cellularity and Schlemm’s canal properties in 11 months age of MYOC^S341P^ transgenic mice (*n* = 3) and WT littermates (*n* = 3). TM trabecular meshwork, SC Schlemm’s canal. **E** Basic anatomy of the mouse eye showing the structure of a representative anterior angle. **F** Hematoxylin and eosin (H&E) staining analysis of the anterior angle structure in MYOC^S341P^ transgenic mice (*n* = 4) and WT littermates (*n* = 4) at 3 and 8 months of age. **G** Slit lamp examination of MYOC^S341P^ transgenic mice (*n* = 4) and WT littermates (*n* = 4) at 3 and 8 months of age. **H** H&E staining analysis of corneal structure in MYOC^S341P^ transgenic mice (*n* = 4) and WT littermates (*n* = 4).

In humans, the S341P MYOC mutation was identified in a pedigree of patients with POAG [35], and it may impair TM function. Next, we performed Gd-MRI to evaluate the aqueous humor dynamics in 3- and 8-month-old MYOC^S341P^ transgenic mice (Tg-MYOC^S341P^) and corresponding age-matched wild-type (WT) mice. Gd signal was gradually enhanced over time and could be used as a tracer for the dynamic accumulation and outflow of aqueous humor. No significant difference in Gd signal was detected between 3-month-old Tg-MYOC^S341P^ and age-matched WT mice, while increased Gd signal enhancement was observed in 8-month-old Tg-MYOC^S341P^ mice relative to the WT group (Fig. 2A, B).

We investigated whether mutant MYOC overexpression elevated IOP in Tg-MYOC^S341P^ mice. Relative to the age-matched WT group, MYOC^S341P^ led to gradual IOP elevation with age, starting at five months of age and continuing until the end of the study (Fig. 2C).

To further explore whether MYOC^S341P^ impairs TM tissue, transmission electron microscopy was performed to assess TM tissue pathology. The results showed reductions in Schlemm’s canal and TM cell loss in 11-month-old Tg-MYOC^S341P^ mice (Fig. 2D). However, no pathological changes were observed in the anterior angle structure or the anterior chamber in Tg-MYOC^S341P^ mice (Fig. 2E–G). Surprisingly, the cornea was also thinner in 11-month-old Tg-MYOC^S341P^ compared to age-matched WT mice (Fig. 2H). These results illustrate that mutant MYOC causes TM tissue dysfunction in vivo.

### RGC loss in MYOC^S341P^ transgenic mice

**Fig. 3:**
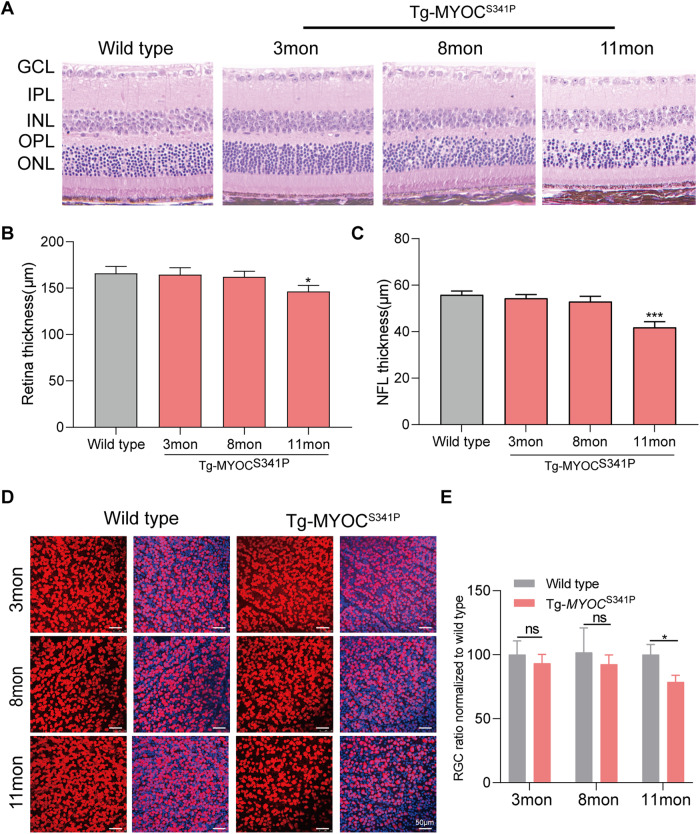
Retinal ganglion cell (RGC) loss in MYOC^S341P^ transgenic mice. **A** Hematoxylin and eosin (H&E) staining of the retina in MYOC^S341P^ transgenic mice (*n* = 12) at 3, 8, and 11 months old and WT littermates at 3 months old (*n* = 3). GCL ganglion cell layer, IPL inner plexiform layer, INL inner nuclear layer, OPL outer plexiform layer, ONL outer nuclear layer. **B** Statistical analysis of retinal thickness experiments illustrated in (**A**). *P < 0.05. **C** Statistical analysis of retinal NFL thickness experiments illustrated in (**A**). NFL: nerve fiber layer. ***P < 0.001. **D** Whole-mount retinal preparations were stained with RBPMS antibody in MYOC^S341P^ transgenic mice and WT littermates at 3, 8, and 11 months of age. **E** Statistical analysis of RGC numbers in MYOC^S341P^ transgenic mice and WT littermates at 3 months (WT: *n* = 6 vs MYOC^S341P^ transgenic mice: *n* = 6), 8 months (WT: *n* = 6 vs MYOC^S341P^ transgenic mice: *n* = 5), and 11 months (WT: *n* = 6 vs *MYOC*^S341P^ transgenic mice: *n* = 5) of age, normalized to the WT group. ns, not significant; *P < 0.05.

To determine whether constant IOP elevation leads to optic neuropathy in the eyes of MYOC^S341P^ transgenic mice, we evaluated retinal morphology, retina thickness, nerve fiber layer (NFL) thickness (GCL + IPL), and the number of RGC in histological sections. The eyes of mutant mice exhibited normal retina morphology, retina thickness, and NFL thickness at 3 and 8 months of age, whereas, at 11 months of age, there were significant reductions in retina and NFL thickness in MYOC^S341P^ transgenic mice (Fig. 3A–C). Hence, cell loss in the NFL and retina thinning were observed in 11-month-old MYOC^S341P^ transgenic mice (Fig. 3A–C). The WT mice used in this experiment were 11 months old. Consistent with these findings, RGC loss was confirmed by flat-mount immunostaining of retinal RBPMS in 11-month-old Tg-MYOC^S341P^ mice (Fig. 3D, E). In contrast, there were no marked changes compared to WT littermates at 3 or 8 months of age (Fig. 3D, E), but at 8 months old, there was a trend toward RGC loss in MYOC^S341P^ transgenic mice (Fig. 3E). These results demonstrate that long-term expression of mutant MYOC leads to RGC loss.

### Impaired visual function in MYOC^S341P^ transgenic mice

**Fig. 4:**
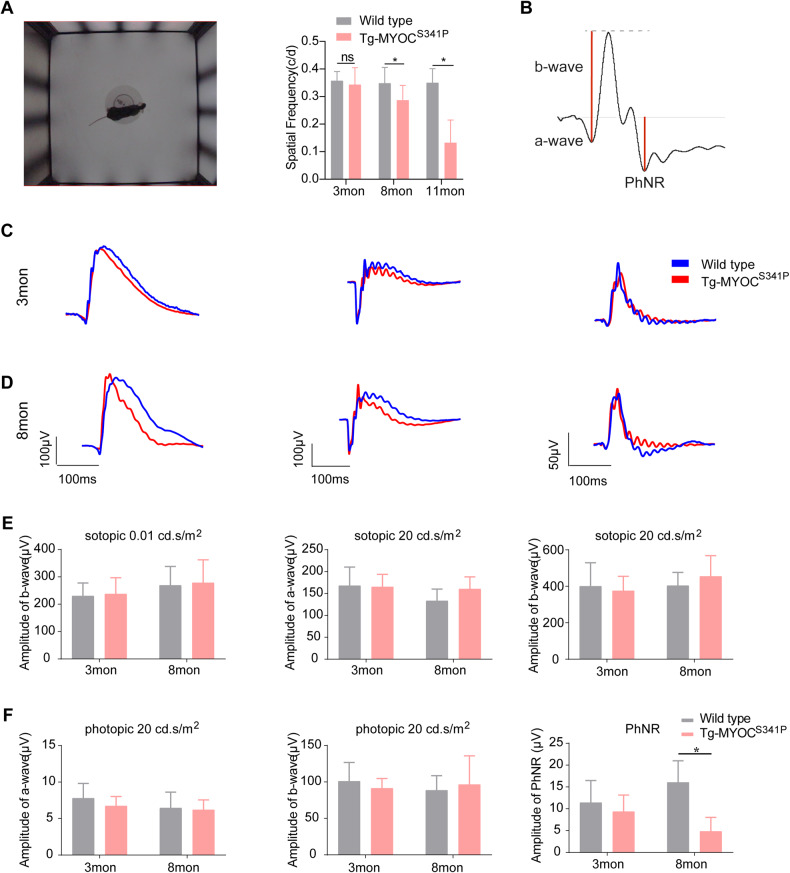
Impaired visual function in MYOC^S341P^ transgenic mice. **A** Schematic diagram of the optomotor response experimental process (left) and the maximum spatial frequency in MYOC^S341P^ transgenic mice and WT littermates aged 3 months (WT: *n* = 12 vs MYOC^S341P^ transgenic mice: *n* = 10), 8 months (WT: *n* = 6 vs MYOC^S341P^ transgenic mice: *n* = 8), and 11 months (WT: *n* = 8 vs MYOC^S341P^ transgenic mice: *n* = 8). ns, not significant; *p < 0.05. **B** Representative a-wave, b-wave, and PhNR in electroretinograms. **C** Waves of scotopic flash stimulation at 0.01 (left), 20 cd.s/m^2^ (middle), and photopic flash stimulation of 20 cd.s/m^2^ (right) in 3-month-old MYOC^S341P^ transgenic mice and age-matched WT littermates. **D** Waves of scotopic flash stimulation at 0.01 (left), 20 cd.s/m^2^ (middle), and photopic flash stimulation of 20 cd.s/m^2^ (right) in 8-month-old MYOC^S341P^ transgenic mice and age-matched WT littermates. **E** Analysis of b-wave under scotopic flash stimulation at 0.01 cd.s/m^2^ (left), a-wave (middle) and b-wave (right) under scotopic flash stimulation at 20 cd.s/m^2^ in 3- and 8-month-old MYOC^S341P^ transgenic mice and age-matched WT littermates. **F** Analysis of a-wave (left), b-wave (middle), and PhNR (right) amplitudes under photopic stimulation at 20 cd.s/m^2^ in 3-and 8-month-old MYOC^S341P^ transgenic mice and age-matched WT littermates. *P < 0.05.

RGC loss would be expected to impair visual function. Therefore, we assessed retinal function by testing optomotor response (OMR) and ERG. A schematic diagram of OMR testing is shown in Fig. 4A. Our results revealed a significant reduction in spatial frequency in 8- and 11-month-old Tg-MYOC^S341P^ mice compared with age-matched WT mice (Fig. 4A). ERG recodes the total charge of the retina after various stimuli and reflects the functional state of the retina. The photopic negative response (PhNR) of ERG is a negative wave following the b-wave, mainly representing the activity of RGCs [36]. Generally, there are no changes in the amplitude of a-wave and b-wave, whereas the amplitude of PhNR is reduced in glaucoma patients. All mice in transgenic and WT groups presented typical a- and b-wave ERG responses (Fig. 4C, D). Stimulus waves, comprising two scotopic flashes (0.01 and 20 cd.s/m^2^) and a 20 cd.s/m^2^ white flash are shown in Fig. 4C and D. No significant differences in a and b-wave amplitude were detected under various stimuli (Fig. 4E, F). Intriguingly, PhNR amplitude was significantly decreased in the 8-month-old Tg-MYOC^S341P^ mice compared to the WT control mice (Fig. 4F). These results indicate that visual function is impaired in MYOC^S341P^ transgenic mice.

### S341P mutant myocilin impairs TM cells

**Fig. 5:**
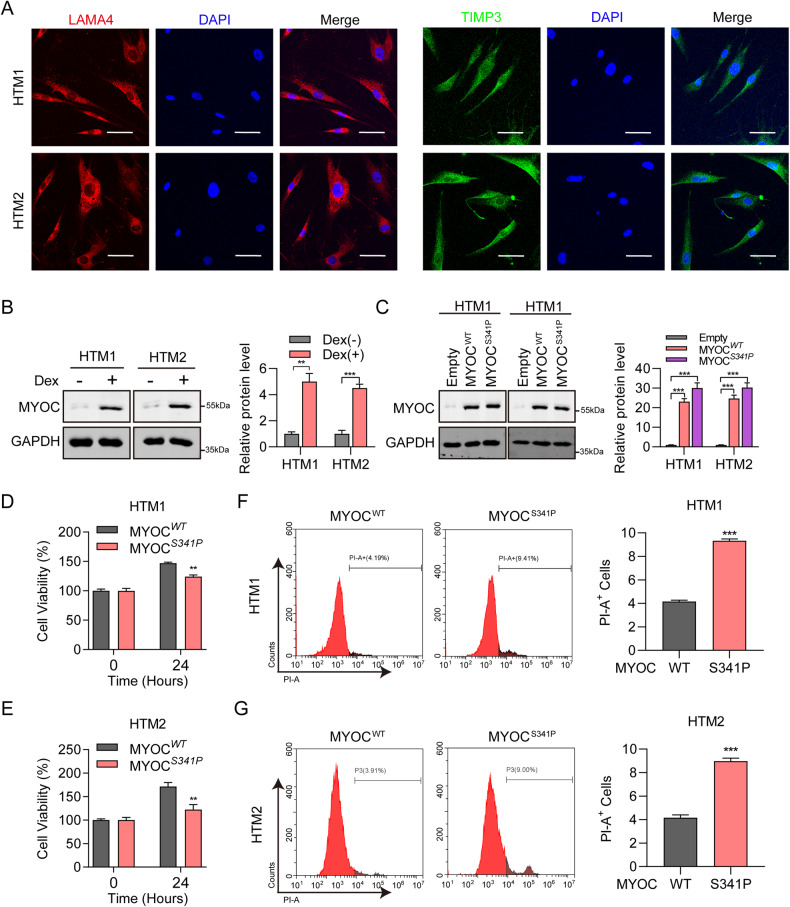
Decreased cell viability and increased cell death in MYOC^S341P^ overexpressing human trabecular meshwork (HTM) cells. **A** Immunofluorescence staining of LAMA 4 (red) and TIMP3 (green) in HTM cells. **B** Western blot detection of MYOC and GAPDH, with or without dexamethasone treatment (left); MYOC band intensity was normalized to that of the untreated group using ImageJ software (right). **p < 0.01; ***p < 0.001. **C** Western blot detection of MYOC and GAPDH in HTM cells overexpressing MYOC^WT^ or MYOC^S341P^ (left); MYOC band intensity was normalized to the empty vector group using ImageJ software (right). ***p < 0.001. Empty, empty vector lentiviruses; MYOC^WT^, lentiviruses expressing the wild-type (WT) myocilin gene; MYOC^S341P^, lentiviruses expressing the S341P mutant myocilin gene. **D**, **E** Cell viability was detected by CCK8 assay in two strains of HTM cells overexpressing MYOC^WT^ or MYOC^S341P^. **p < 0.01. MYOC^WT^, lentiviruses expressing the WT myocilin gene; MYOC^S341P^, lentiviruses expressing the S341P mutant myocilin gene. **F** Flow cytometry analysis of propidium iodide (PI)-positive cells in HTM1 after MYOC^WT^ or MYOC^S341P^ overexpression (left). Statistical analysis of the proportion of positive cells (right). ***p < 0.001. MYOC^WT^, lentiviruses expressing the WT myocilin gene. MYOC^S341P^, lentiviruses expressing the S341P mutant myocilin gene. **G** Flow cytometry analysis of PI-positive cells in HTM2 after MYOC^WT^ or MYOC^S341P^ overexpression (left). Statistical analysis of the proportion of positive cells (right). ***p < 0.001. MYOC^WT^, lentiviruses expressing the WT myocilin gene. MYOC^S341P^, lentiviruses expressing the S341P mutant myocilin gene.

To investigate the detailed mechanism of MYOC^S341P^, we used two strains of human primary TM cells for in vitro studies. The strains were characterized by laminin subunit alpha 4 (LAMA4) staining, TIMP metallopeptidase inhibitor 3 (TIMP3) staining, and Dex treatment. LAMA4 and TIMP3 exhibited strong fluorescence intensity (Fig. 5A), and myocilin expression was robustly induced at the protein level after Dex treatment (Fig. 5B). To detect biological function, TM cells were transduced with lentivirus expressing MYOC^WT^ or MYOC^S341P^, which were successfully overexpressed (Fig. 5C). Compared with MYOC^WT^, MYOC^S341P^ inhibited viability (Fig. 5D, E) and promoted cell death (Fig. 5F, G) of the two TM cell strains.

### S341P mutant myocilin induces ER stress

**Fig. 6:**
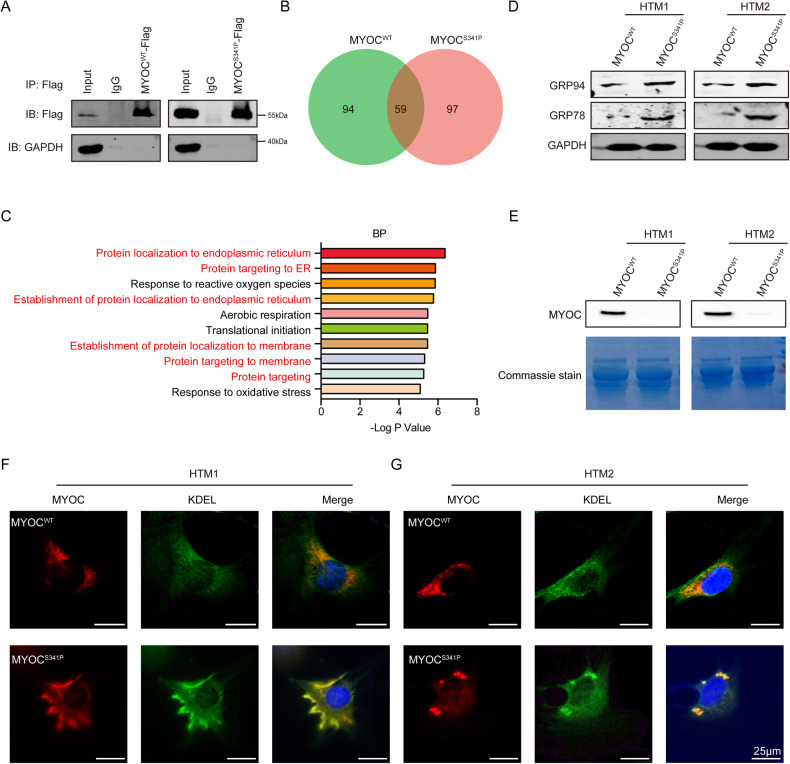
Induced ER stress in HTM cells overexpressing MYOC^S341P^. **A** Western blot analysis of MYOC^WT^ or MYOC^S341P^ after co-immunoprecipitation. **B** Analysis of proteins differentially interacting with MYOC^WT^ and MYOC^S341P^. **C** Analysis of the biological functions of proteins differentially interacting with MYOC^WT^ and MYOC^S341P^. **D** Western blot detection of GRP78, GRP94, and GAPDH in two strains of HTM cells overexpressing MYOC^WT^ or MYOC^S341P^. MYOC^WT^, lentiviruses expressing the wild-type (WT) myocilin gene. MYOC^S341P^, lentiviruses expressing the S341P mutant myocilin gene. **E** Western blot detection of MYOC in two strains of HTM cells overexpressing MYOC^WT^ or MYOC^S341P^ (upper). Coomassie staining analysis of total proteins in two strains of HTM cells overexpressing MYOC^WT^ or MYOC^S341P^ (lower). MYOC^WT^, lentiviruses expressing the WT myocilin gene. MYOC^S341P^, lentiviruses expressing the S341P mutant myocilin gene. **F**, **G** Immunofluorescence staining of MYOC (red) and KDEL (green) in two strains of HTM cells overexpressing MYOC^WT^ or MYOC^S341P^.

Proteins function together with other interacting proteins. Hence, we applied immunoprecipitation followed by mass spectrometry (IP-MS) to identify proteins that differentially interacted with MYOC^WT^ and MYOC^S341P^. Successful IP was verified by western blot (Fig. 6A). Fifty-nine proteins overlapped between MYOC^WT^ and MYOC^S341P^, and 97 proteins differentially interacted with MYOC^S341P^ (Fig. 6B and Table S1). Biological process analysis indicated that proteins that differentially interacted with MYOC^S341P^ were enriched for localization to the ER and targeting of the ER (Fig. 6C).

Based on the above analysis, we analyzed levels of ER stress-related proteins and found Grp 78 and Grp 94 were significantly upregulated in the MYOC^S341P^ group (Fig. 6D), consistent with the results in vivo (Fig. 1F, G). Moreover, mutant MYOC failed to be secreted into the cellular supernatant (Fig. 6E). Consistent with these findings, MYOC^S341P^ was found to colocalize with ER-resident proteins and aggregated intracellularly by immunofluorescence analysis (Fig. 6F, G).

Moreover, we applied 4-PBA to promote the secretion of mutant MYOC and confirm whether non-secretion of mutant MYOC is the main cause of TM cells injury. As shown in Fig. S1A, 4-PBA promoted mutant MYOC secretion. Additionally, 4-PBA treatment partially reversed cell viability (Fig. S1B) and alleviated cell death (Fig. S1C) in TM cells with MYOC^S341P^ overexpression.

These results indicate that the non-secretion of mutant MYOC induces ER stress.

### S341P mutant myocilin causes autophagy dysfunction

**Fig. 7:**
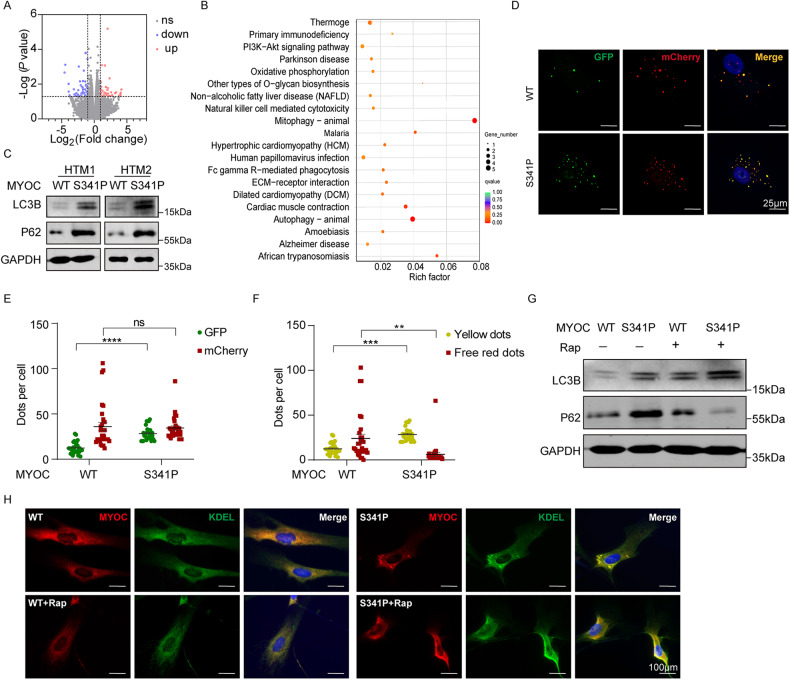
Impaired autophagy in HTM cells overexpressing MYOC^S341P^. **A** RNA-seq analysis of genes differentially expressed between MYOC^WT^ and MYOC^S341P^. **B** KEGG analysis of proteins differentially expressed between MYOC^WT^ and MYOC^S341P^. **C** Western blot detection of LC3B, P62, and GAPDH in two strains of HTM cells overexpressing MYOC^WT^ or MYOC^S341P^. WT, lentiviruses expressing the wild-type (WT) myocilin gene. S341P, lentiviruses expressing the S341P mutant myocilin gene. **D**–**F** HTM cells were transfected with lentiviruses expressing MYOC^WT^ or MYOC^S341P^ for 48 h, then transduced with Ad5-mCherry-GFP-LC3 for 24 h. GFP dots, mCherry dots, yellow dots, and free mCherry dots were counted. **p < 0.01; ***p < 0.001, ****p < 0.0001. WT, lentiviruses expressing the WT myocilin gene. S341P, lentiviruses expressing the S341P mutant myocilin gene. **G** Western blot detection of LC3B, P62, and GAPDH in HTM cells overexpressing MYOC^WT^ or MYOC^S341P^, with or without rapamycin treatment. WT, lentiviruses expressing the WT myocilin gene; S341P, lentiviruses expressing the S341P mutant myocilin gene. **H** Immunofluorescence staining of MYOC (red) and KDEL (green) in two HTM cells overexpressing MYOC^WT^ or MYOC^S341P^ without Rap treatment (upper) or with Rap treatment (lower).

To investigate the underlying molecular mechanism further, we conducted an RNA-seq assay to assess differentially expressed downstream genes in the MYOC^S341P^ group. Numbers and trends in DEGs are illustrated in Fig. 7A. Pathways enriched for DEGs are presented in Fig. 7B, and the results indicated that autophagy might participate in MYOC^S341P^-induced TM cell impairment.

We observed impaired autophagy in the MYOC^S341P^ group, evidenced by increased levels of p62 and LC3B (Fig. 7C), consistent with the results in vivo (Fig. 1F, G). mCherry-GFP-LC3 is widely used to monitor autophagic flux because of the difference in stability of GFP and mCherry fluorescent proteins at variable pH [37]. After infection with lentivirus expressing MYOC^WT^ or MYOC^S341P^, human primary TM cells were transduced with mCherry-GFP-LC3 adenovirus for 24 h. The results demonstrated that the number of GFP dots was upregulated, while there was no significant change in mCherry dots in the mutant myocilin group (Fig. 7D, E). Consistently, yellow puncta were increased, while free red puncta were reduced significantly in the mutant myocilin group (Fig. 7F), implying reduced autophagic flux. To further validate the role of autophagy, we stimulated autophagy using rapamycin (Rap). As indicated in Fig. 7G, autophagy was significantly activated, as verified by increased LC3B and decreased P62 protein levels in the WT group after Rap treatment (Fig. 7G). As expected, de novo activation of autophagy relieved mutant MYOC accumulation (Fig. 7H). These results demonstrate that deregulated autophagy participates in mutant MYOC-associated TM impairment.

## Discussion

Elevation in IOP secondary to TM injury is the primary cause of POAG. Studies on genetic factors related to TM impairment are scarce. To date, few genes have been validated as associated with TM dysfunction, and MYOC was the first gene identified as causal for POAG. The S341P MYOC mutation was identified in a pedigree of patients with POAG and is associated with elevated IOP [35]. However, more insight is needed into whether MYOC^S341P^ leads to glaucoma in vivo. Moreover, the function and mechanism underlying the effects of MYOC^S341P^ in TM biology require exploration. In this study, we developed a MYOC^S341P^ transgenic mouse model, which closely mimicked the phenotypes observed in patients with POAG, including elevated IOP, RGC loss, and visual impairment. MYOC^S341P^ overexpression caused TM dysfunction, which was depicted as decreased aqueous humor outflow, loss of TM cells in MYOC^S341P^ transgenic mice, and reduced cell viability in vitro. Mechanistically, dysregulated autophagy may be involved in this process.

Mouse models that exhibit duplicate phenotypes with high IOP POAG patients are insufficient. Increasing IOP by obstructing aqueous humor outflow or laser treatment of the TM may introduce unexpected variations of induced inflammation and foreign body immune reaction. Genetically based glaucoma models are more advantageous. In this study, we developed a MYOC^S341P^ transgenic mouse model that has glaucoma characteristics. It was reported that MYOC^WT^ transgenic mice did not exhibit glaucoma phenotypes [38], so it is speculated that the pathological phenotypes in POAG patients are only correlated with abnormal mutant MYOC proteins. It is widely recognized that studies investigating the pathological mechanism of mutant MYOC proteins in vivo applied C57BL/6J strain to serve as a control [16, 39, 40]. Therefore, we used C57BL/6J strain as a WT control.

Elevated IOP is a major risk factor for glaucoma. We found that IOP increased in MYOC^S341P^ transgenic mice from 5 months of age until the end of the study. Long-term IOP elevation resulted in TM dysfunction and injury, evident from aqueous humor dynamics and TM cell loss in MYOC^S341P^ transgenic mice. RGC damage is another complication of long-term elevations in IOP. In this study, RGC loss was observed in 11-month-old MYOC^S341P^ transgenic mice, while there was no marked difference between WT and MYOC^S341P^ transgenic mice at 8 months of age—after seven months of IOP increase. However, there was a trend toward thinning of the GCL. Furthermore, at 8 months of age, PhNR amplitude and OMR were reduced, indicating that RGC function was impaired at 8 months of age, although there were no significant reductions in the number of RGCs.

An effective strategy to understand the difference in function between MYOC^S341P^ and MYOC^WT^ is to identify differentially interacting proteins since interacting proteins generally act in similar roles and the same physiological processes and pathologies [41]. Previous studies have confirmed the interaction of MYOC^WT^ with Hevin [42], sFRP1, sFRP3 [43], syntrophins [44], gliomedin, neurofascin, and NrCAM [45], indicating its roles in the central nervous system, skeletal muscle, and development. However, investigation of proteins interacting with mutant myocilin and comparisons of proteins differentially interacting with WT and mutant myocilin are scarce. In this study, we performed IP-MS to identify proteins that interacted with MYOC^WT^ or MYOC^S341P^. By clustering differentially interacting proteins, we systemically integrated the functional differences between MYOC^WT^ and MYOC^S341P^. According to our IP-MS results, response to reactive oxygen species and response to oxidative stress were also among the top ten biological processes detected by clustering proteins that differentially interacted with MYOC^S341P^. Consistent with the IP-MS findings, RNA-seq analysis showed that, besides autophagy, response to hydrogen peroxide, hydrogen peroxide catabolic process, and hydrogen peroxide metabolic process were also among the most enriched biological processes in the MYOC^S341P^ group, which are all involved in oxidative stress. Oxidative stress is one of the major changes in TM related to glaucoma [46]. Under low-grade oxidative stress, autophagy is activated and autophagic flux is normal, whereas autophagic flux is impaired in TM cells under high-grade oxidative stress [47]. Hence, we speculate that there is an interplay between autophagy and oxidative stress. The deregulated autophagy observed in MYOC^S341P^ may result from the interplay between accumulated mutant MYOC and oxidative stress. The role of mutant myocilin in oxidative stress has been reported in a previous study, which demonstrated that different mutant myocilin behave different sensitivity to oxidative stress [48]. The interplay of autophagy and oxidative stress in MYOC^S341P^ caused by TM dysfunction needs further investigation.

Autophagy is stimulated to cope with various intracellular and extracellular stress and maintain cellular homeostasis [49]. TM cells cannot dilute undesirable protein aggregation by cell division owing to their limited proliferative potential [9]. Hence, the TM is particularly reliant on autophagic pathways. Numerous studies have indicated that autophagy has an indispensable role in the TM. Autophagy dysregulation in the TM has been validated in a high IOP model of glaucoma [50]. Further, autophagy is triggered to maintain IOP homeostasis in response to constant physiological and pathological stretch [51–53]. Herein, we found that mutant myocilin caused autophagy dysfunction, illustrated by impeded autophagic flux. Additionally, the activation of autophagy by Rap alleviated intracellular accumulation of mutant MYOC proteins. We speculate that autophagy dysregulation may contribute to the TM dysfunction in MYOC^S341P^ transgenic mice, eventually causing glaucoma development. The detailed relationship between autophagy and MYOC^S341P^ transgenic mice warrants further study. Autophagy also has important roles in other neurodegenerative diseases [49]. Therefore, clarifying the effects of autophagy in glaucoma may benefit research into other serious neurodegenerative conditions, and autophagy stimulating strategies have huge potential for treatment of neurodegenerative diseases. Because of the lack of clinical specimen, the results observed from the in vitro and in vivo studies fail to validate in patients with POAG.

Our study provides evidence that can form the basis for further investigation of the functional differences between WT and mutant MYOC. Additionally, it renders an effective transgenic model for mechanistic studies and the screening of therapeutic targets for glaucoma.

## Materials and methods

### Generation of a transgenic mouse model by CRISPR/Cas 9

This study was approved and monitored by the Institutional Animal Care and Use Committee of the Capital Medical University of Beijing (IACUC; AEEI-2018-198) and conformed to the National Institute of Health Guide for the Care and Use of Laboratory Animals, as well as the Association for Research in Vision and Ophthalmology Statement for the Use of Animals in Ophthalmic and Vision Research.

The method for constructing S341P MYOC transgenic mice was similar to that described in our previous article [15]. In brief, an S341P mutation was introduced in WT MYOC cDNA using a Quick Change Site-Directed Mutagenesis kit (Stratagene). The CRISPR/Cas9 system was used to introduce S341P MYOC into the H11 locus of fertilized C57BL/6J mouse zygotes by homologous recombination. Mice were genotyped by PCR and Sanger sequencing, and F3 and later generations were used in this study.

### IOP measurement

Animals were anesthetized with isoflurane, and 15 measurements were acquired using TonoLab rebound tonometry (Icare, Helsinki, Finland). IOP was measured between 3:00 and 5:00 p.m.

### Evaluation of aqueous humor dynamics

Gadolinium magnetic resonance imaging (Gd-MRI) was applied to evaluate aqueous humor dynamics. Mice were anesthetized with 2% isoflurane, and respiration rate was monitored using a small pneumatic pillow. Baseline measurements were initially obtained, and then mice were injected intraperitoneally with 0.3 mmol/kg gadolinium-DTPA. Images were acquired every 10 min for 1 h. Signal intensity in areas of interest was analyzed using ImageJ software, and all measurements were normalized to the baseline image.

### Electroretinography

An Espion Visual Electrophysiology System (Diagnosys, USA) was used to record electroretinograms (ERGs). After at least 12 h of dark adaptation, animals were anesthetized, and their pupils dilated using 0.2 mg/mL tropicamide phenylephrine. Animals were placed on a regulated heating pad throughout the experiment. ERGs were recorded using a golden ring that made contact with the corneal surface through a 0.2% carbomer layer. Additionally, needle electrodes were inserted into the cheeks and tails of the animals to serve as reference and ground leads, respectively. The ERG stimulus consisted of two scotopic flashes (0.01 and 20 cd.s/m^2^), followed by 20 cd.s/m^2^ white flashes on a 30 cd/m^2^ white background after a light preadaptation period of 5 min. A-waves, b-waves, and PhNR were recorded and analyzed. PhNR refers to a negative wave following the b-wave that mainly originates from the spiking activity of RGCs and amacrine cells [54]. The amplitudes of different wave types from each eye were recorded for analysis. Amplitudes of a- and b-waves are reported as mean ± standard deviation. Statistical analysis was performed using GraphPad Prism software. A multiple *t*-test was used to compare the two groups. A *p*-value < 0.05 was considered significant.

### Retinal flat mounts

Enucleated eyes were immersed in 4% PFA for 2 h at 4 °C, followed by permeabilization with 0.3% Triton-X100 for 3 h at room temperature and blocking in 1% bovine serum albumin (BSA) for 2 h at room temperature. Then, retinas were incubated with RBPMS antibody (GTX118619, GeneTex) overnight at 4 °C. The next day, retinas were returned to room temperature and incubated with Alexa Fluor 546-conjugated secondary antibody (A10036; Thermo) for 1 h at room temperature. Nuclei were stained with DAPI (Vector Laboratories, Peterborough, United Kingdom). Images were captured with a Leica confocal imaging system, and ImageJ was used to count RGCs.

### Optomotor response (OMR)

A virtual optomotor tracking system (OptoMotry, Lethbridge, Canada) was applied to evaluate visual function. Mice were placed in the middle of the platform and allowed to adapt to the environment for 2 min. Then, mice were stimulated with different grating frequencies and monitored using an overhead camera, and the grating frequency was varied in a stepwise manner. Grafting rotation, in either a clockwise or counterclockwise direction, was used to assess the visual function of left or right eyes. Mean spatial frequency values were recorded and analyzed.

### Cell culture

Human primary TM cells were obtained from ScienCell (Carlsbad, CA, United States, catalog: 6590, 5987) and grown in TMCM medium (ScienCell), supplemented with 2% fetal bovine serum (ScienCell), 1% penicillin/streptomycin solution (ScienCell), and 1% TM cell growth supplement (ScienCell). All cells used for experiments were cultured for < 6 passages. TM cell characterization was performed as previously described [55]. Briefly, TM cells were treated with 100 nM dexamethasone (Dex, Selleck) for 7 days to specify the origin of cell strains.

### Lentivirus treatment

For lentivirus transfection, 1.0 × 10^5^ TM cells were seeded into 6-well plates and cultured for approximately 12 h. Lentivirus expressing WT or S341P MYOC-infected TM cells were added at a multiplicity of infection of 5. Polybrene (5 μg/mL) was used to enhance lentivirus infection efficiency. Culture medium was exchanged for fresh medium within 24 h. Cells were harvested for subsequent analysis at the indicated time points.

### Western blot analysis

TM cells were lysed in RIPA buffer (P0013B, Beyotime) for 30 min on ice. Protein concentrations were quantified using a BCA kit (23227, Thermo). Total protein aliquots (40 μg) were subjected to 10% sodium dodecyl sulfate-polyacrylamide gel electrophoresis and transferred to the PVDF membrane (Millipore). Membranes were blocked in 5% non-fat milk for 2 h, then incubated with primary antibodies at 4 °C overnight. After washing three times with 0.1% TBST, membranes were incubated with appropriate secondary antibodies. Signals were detected using an ODYSSEY Clx system. The following antibodies were used: myocilin (sc-515500, sc-137233, Santa Cruz Biotechnology), GAPDH (sc-25778, Santa Cruz biotechnology), Grp78 (3177, Cell Signaling Technology), Grp 94 (2104, Cell Signaling Technology), p62 (5114, Cell Signaling Technology), LC3B (43566, Cell Signaling Technology), IRDye 800CW goat anti-rabbit IgG (926-32211, LI-COR), and IRDye 800CW goat anti-mouse IgG (926-32210, LI-COR).

### Cell viability detection

TM cells transfected with WT or S341P MYOC lentivirus were seeded into 96-well plates in triplicate at a density of 1000 cells per well for 24 h. CCK-8 (10 μL) solution was added to cells. The cells were then incubated for 2 h, and cell viability was determined by measuring absorbance at 450 nm using a microplate reader.

### Cell death detection

TM cells were digested using EDTA-free trypsin for 1 min, then centrifuged for 5 min at 300 g. The cells were then washed twice with pre-cooled PBS. Cells were resuspended in 1× binding buffer at a final concentration of 2 × 10^6^/mL. Propidium iodide staining solution (10 μL) and PBS (400 μL) were added to the cells, and flow cytometry was immediately conducted to analyze cell death.

### Immunoprecipitation (IP)

TM cells transfected with flag-tagged WT or S341P MYOC were cultured in 10 cm culture dishes and harvested when they reached 90% confluence. Cells were lysed in IP lysis buffer (20 mM Tris (pH7.5), 150 mM NaCl, 1% Triton X-100), containing proteinase inhibitor, on ice for 5 min with periodic agitation. Cell lysates were centrifuged, and supernatants were collected. Supernatant aliquots (80 μL) served as input samples, and the remaining supernatants were equally divided into samples for analysis of IgG and the protein of interest, incubated at 4 °C overnight with rotation with either IgG or Flag antibody-protein A/G magnetic beads, respectively. Antibody-protein A/G magnetic beads complexes were washed with PBST. RIPA buffer was then added, and beads were boiled for 5 min at 95 °C. Antibody-protein A/G magnetic bead complexes were magnetically separated for 3 min, and the supernatants were used for subsequent western blot analysis.

### IP followed by mass spectrometry (IP-MS)

Protein complex samples collected by immunoprecipitation were preprocessed and analyzed by liquid chromatography-mass spectrometry (MS). Briefly, LC-MS/MS was performed using a Q-Exactive mass spectrometer coupled with an Easy nLC (Thermo Fisher Scientific, MA, USA). The peptide sample was first loaded onto the C18-reversed phase analytical column (Thermo scientific, Acclaim PepMap RSLC 50 μm × 15 cm, nano viper, P/N164943) in buffer A (0.1% formic acid in HPLC grade water), and separated with a linear gradient of buffer B (80% acetonitrile and 0.1% Formic acid) with a flow rate at 300 nL/min. The linear chromatographic gradient was achieved with a linear increase of buffer B percentage, which was set up as follows: 6% buffer B for 5 min, 6%–28% buffer B for 40 min, 28%–38% buffer B for 5 min, 38%–100% buffer B for 5 min, and held in 100% buffer B for 5 min. After that, the peptide entered into the Q-Exactive mass spectrometer (Thermo Fisher Scientific, MA, USA). The MS analysis was set for 60 min in a positive ion mode. MS data was acquired using a data-dependent top 10 method, dynamically choosing the most abundant precursor ions from the full scan (350–1800 *m/z*) for HCD fragmentation. Full scans were acquired at a resolution of 70,000 at *m/z* 200 with an AGC target of 3e6 and a maxIT of 50 ms. MS2 scans were acquired at a resolution of 17500 for HCD spectra at m/z 200 with an AGC target of 2 × 10^5^ and a maxIT of 45 ms. The scan range was 200–2000 *m/z*, and the isolation width was 2 *m/z*. Only ions with a charge state between 2–7 and a minimum intensity of 2 × 10^3^ were selected for fragmentation. Dynamic exclusion for selected ions was 30 s, and the microscan was 1. The normalized collision energy was 27 eV. The raw data obtained was then imported into Proteome Discoverer 2.2 (Thermo Fisher Scientific) for protein identification, and then embedded Mascot 2.6 engines were used for database searches. Kyoto Encyclopedia of Genes and Genomes (KEGG) pathway enrichment analysis was conducted using the “clusterprofiler” package in R language.

### Immunofluorescence

TM cells were plated on coverslips in 24-well plates and grown overnight to 50%–60% confluence. Cells were fixed with 4% paraformaldehyde (PFA) for 12 min at room temperature and permeabilized with 0.3% Triton X-100 (CWBIO) for 15 min. Subsequently, cells were blocked with 2% BSA for 1 h at room temperature. Then, cells were incubated with primary antibodies at 4 °C overnight, followed by incubation with secondary antibodies for 1 h. Finally, cells were stained with 4,6-diamidino-2-phenylindole (DAPI). Fluorescence images were captured using Zeiss or Leica confocal imaging systems (Carl Zeiss; Leica). The following antibodies were used: p62 (5114, Cell Signaling Technology), LC3B (43566, Cell Signaling Technology), KDEL (ab176333, Abcam), RBPMS (GTX118619, GeneTex), Alexa Fluor 488 (A21206; Thermo), and Alexa Fluor 546 (A10036; Thermo).

### Treatment with 4-Phenylbutyrate (4-PBA)

Dimethyl sulfoxide (DMSO; Thermo) or PBA (1 mM; Thermo) was added to the culture medium of TM cells overexpressing MYOC^WT^ or MYOC^S341P^ for 48 h. The culture medium containing DMSO or PBA was replaced daily.

### RNA extraction and sequencing

RNA was extracted using Trizol reagent (Thermo) according to the manufacturer’s instructions. RNA purity was assessed using a NanoPhotometer spectrophotometer (IMPLEN, CA, USA), and RNA integrity was evaluated using an RNA Nano 6000 Assay Kit on the Agilent Bioanalyzer 2100 system (Agilent Technologies, CA, USA). RNA samples (2 μg) were used as input material for the preparation of sequencing libraries using a NEBNext Ultra^TM^ RNA Library Prep Kit for Illumina (NEB, USA) according to the manufacturer’s instructions. After cluster generation, prepared libraries were sequenced on an Illumina Hiseq 4000 platform to generate paired-end 150-bp reads. After quality control, high-quality reads were mapped to the reference genome sequence. Only reads with a perfect match or a single base pair mismatch were further analyzed and annotated based on the reference genome.

Differential expression analysis was performed using the DESeq package in R (1.10.1). The resulting *P*-values were adjusted using the Benjamini and Hochberg approach to control the false discovery rate. Genes with an adjusted *p*-value < 0.05 by DESeq were assigned as differentially expressed.

Functional analysis and KEGG pathway analysis of the annotated list of differentially expressed genes (DEGs) were conducted using the GOseq R package and KOBAS software.

### Autophagic flux measurement

Autophagic flux was detected using an mCherry-GFP-LC3 tandem fluorescent protein quenching assay. TM cells transfected with WT or S341P MYOC lentivirus for 48 h were seeded into 24-well chamber slides and re-transduced with mCherry-GFP-LC3 adenovirus for a further 24 h. The cells were fixed in 4% PFA for 10 min, and nuclei were stained with DAPI (Vector Laboratories, Peterborough, United Kingdom). Fluorescent images were captured immediately using a Zeiss microscope.

### Drug treatment

Rapamycin was used to activate autophagy. After transfection with WT or S341P MYOC lentivirus for 48 h, TM cells were treated with rapamycin (10 μM) for 48 h before harvesting for analysis of autophagy-related proteins. Furthermore, MYOC and KDEL colocalization were analyzed by immunofluorescence.

### Statistical analysis

Statistical data are reported as mean ± standard error of the mean of at least three independent biological repeats. Statistical analysis was performed using GraphPad Prism software. The Student’s *t*-test was used to compare two groups, and one-way analysis of variance (ANOVA) was used for comparisons of more than two groups. A *p*-value < 0.05 was considered significant.

### Reporting summary

Further information on research design is available in the Nature Research Reporting Summary linked to this article.

### Supplementary information

Table S1

Fig S1

Reporting Summary

WB org

## Data Availability

Data supporting the present study are available from the corresponding author upon reasonable request.
