# Supplementary material for: Serine to proline mutation at position 341 of MYOC impairs trabecular meshwork function by causing autophagy deregulation
Source: Cell Death Discov. 2024 Jan 11;10:21. doi: 10.1038/s41420-024-01801-1 (PMC10784477; doi:10.1038/s41420-024-01801-1)
Supplement: Supplementary file 2 — Fig S1 [file 41420_2024_1801_MOESM2_ESM.docx]

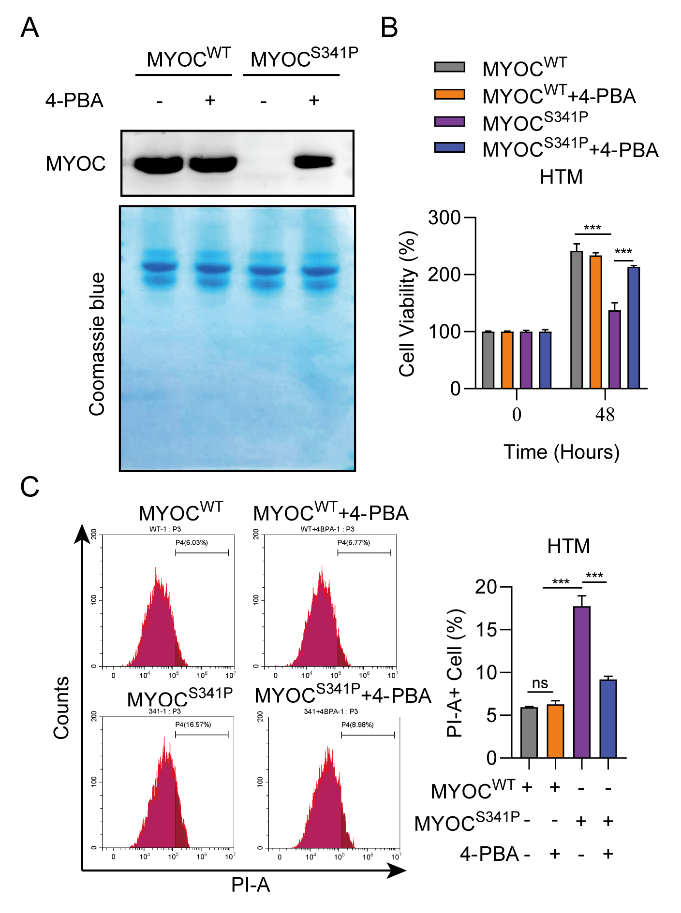


**Fig. S1** Relieving ER stress rescues cell viability and cell death in TM cells

(A) Western blot analysis of MYOC in cell supernatant in MYOC^WT^ or MYOC^S341P^ with or without 4-PBA treatment.

(B) Cell viability analysis of TM cells overexpressing MYOC^WT^ or MYOC^S341P^ with or without 4-PBA treatment. ***p < 0.001.

(C) Cell death analysis of TM cells overexpressing MYOC^WT^ or MYOC^S341P^ with or without 4-PBA treatment. ns, not significant; ***P < 0.001.
