# Supplementary figures and images for: Serine to proline mutation at position 341 of MYOC impairs trabecular meshwork function by causing autophagy deregulation
Source: Cell Death Discov. 2024 Jan 11;10:21. doi: 10.1038/s41420-024-01801-1 (PMC10784477; doi:10.1038/s41420-024-01801-1)

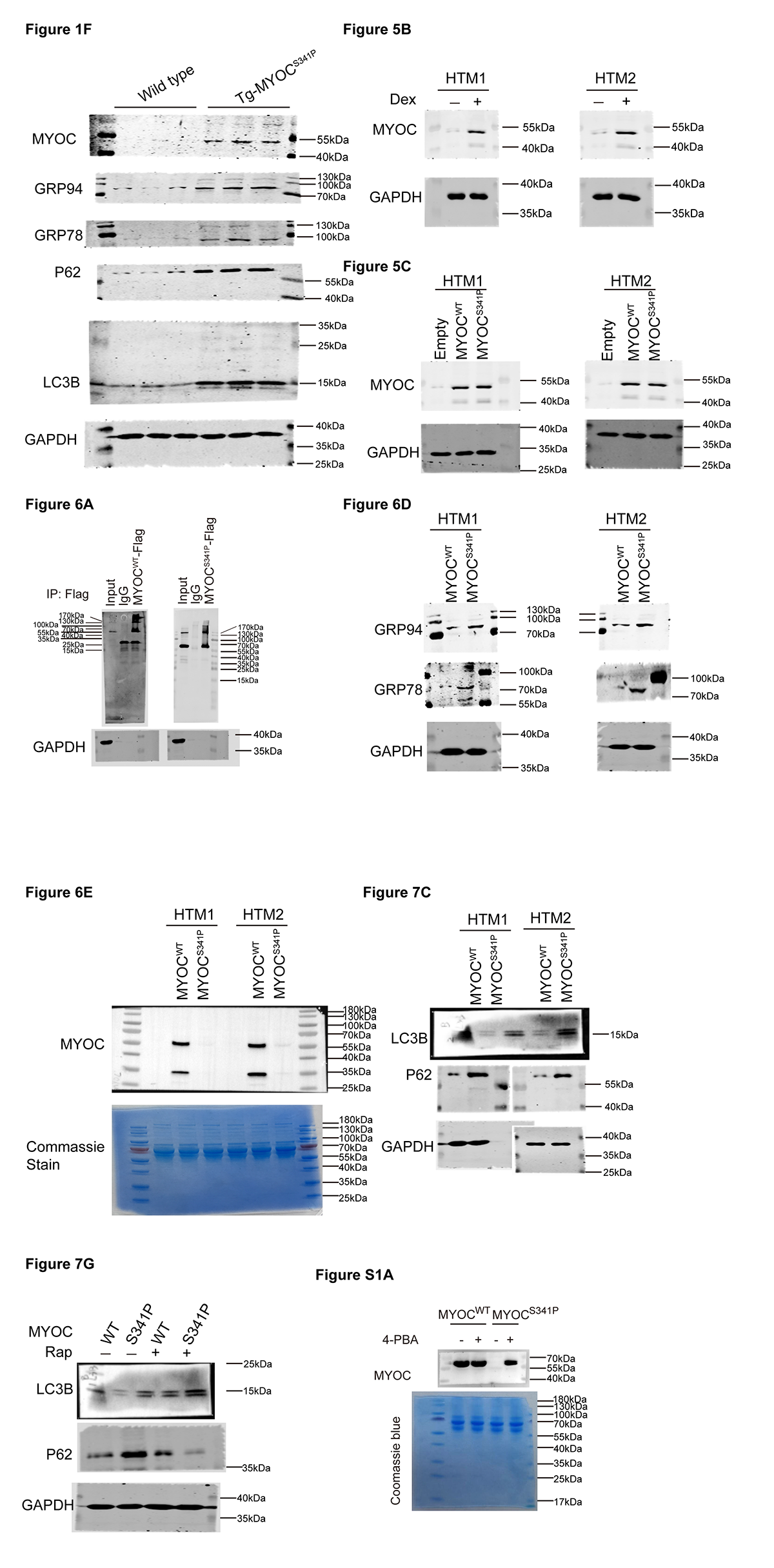

Supplement: Supplementary file 4 — WB org [file 41420_2024_1801_MOESM4_ESM.tif]
